# Nucleotides and AHCC Enhance Th1 Responses In Vitro in *Leishmania*-Stimulated/Infected Murine Cells

**DOI:** 10.3390/molecules25173918

**Published:** 2020-08-27

**Authors:** María Auxiliadora Dea-Ayuela, Sergi Segarra, Dolores R. Serrano, Francisco Bolás-Fernández

**Affiliations:** 1 Departamento de Farmacia, Facultad de Ciencias de la Salud, Universidad CEU-Cardenal Herrera, 46113 Moncada, Spain; mda_3000@yahoo.es; 2 R & D Bioiberica S.A.U., Av. dels Països Catalans 34, 08950 Esplugues de Llobregat, Spain; 3 Department of Pharmaceutics and Food Technology, School of Pharmacy, Universidad Complutense de Madrid, Plaza Ramon y Cajal s/n, 28040 Madrid, Spain; dr.serrano@farm.ucm.es; 4 Instituto Universitario de Farmacia Industrial (IUFI), School of Pharmacy, Universidad Complutense de Madrid, Avenida Complutense, 28040 Madrid, Spain; 5 Departament of Microbiology and Parasitology, School of Pharmacy, Universidad Complutense de Madrid, Plaza Ramon y Cajal s/n, 28040 Madrid, Spain; francisb@farm.ucm.es

**Keywords:** nucleotides, AHCC, *Leishmania* spp, Th1 response, cytokines, promastigotes, amastigotes, mechanism of action

## Abstract

A stronger Th1 (cellular) immune response in canine leishmaniosis (CanL) leads to a better prognosis. Dietary nucleotides plus AHCC^®^ have shown beneficial effects in dogs with clinical leishmaniosis and in clinically healthy *Leishmania*-infected dogs. The potential leishmanicidal activity of nucleotides and AHCC was assessed by quantifying nitric oxide (NO) production and replication of parasites. Their effects on lymphocyte proliferation were studied with and without soluble *Leishmania infantum* antigen (SLA) stimulation. Cytokine level variations were assessed using naïve and *L. infantum*-infected macrophages/lymphocytes cocultures. Promastigotes and amastigotes proliferation and NO macrophage production were not directly affected. Lymphocyte proliferation was significantly enhanced by nucleotides, AHCC, and their combinations only after SLA stimulation. Nucleotides and AHCC significantly increased the production of IL-1β, IL-2, IL-5, IL-9, IL-10, and IL-12 by naïve immune cells. In naïve and *L. infantum*-infected macrophage/lymphocyte cocultures, nucleotides with or without AHCC led to significant increases in IFN-γ and TNF-α. Given that these cytokines are involved in the effective Th1 immune response against *Leishmania* parasites, these mechanisms of action could explain the previously reported in vivo clinical efficacy of such combination and further support the use of nucleotides with or without AHCC in the management of CanL patients.

## 1. Introduction

Unicellular flagellates of the family Trypanosomatidae are obligatory parasites of invertebrates, vertebrates, and plants. This family includes pathogens responsible for African sleeping sickness, Chagas’ disease, and leishmaniases, among others. The subfamily Leishmaniinae includes monoxenous parasites of insects and dixenous parasites of insects and vertebrates (genera *Leishmania*, *Paraleishmania* and *‘Endotrypanum’*) [1,2]. Leishmaniasis is a complex of infectious diseases caused by protozoan parasites belonging to species of the genus *Leishmania*. These diseases affecting humans and several wild and domestic mammals are transmitted by Diptera arthropods belonging to genus *Lutzomyia* in the New World or *Phlebotomus* in the Old World [3]. The clinical spectrum of the disease is largely dependent on the infecting species and the intrinsic characteristics of the host, varying from the benign cutaneous forms to the most severe form, visceral leishmaniasis, which is fatal if left untreated [4].

In urban areas, domestic dogs are the main parasite reservoir which effectively takes part in its transmission. The decision on whether or not to treat seropositive dogs is still under debate given that treatment can be toxic to animals and generate drug resistances, which may be extended to humans. Very few drugs are available at the clinical level; therefore, the search for new therapies for both humans and animals is an urgent task [5]. Pentavalent antimonials (SbV) are the current first-choice drugs prescribed by veterinary practitioners against canine leishmaniosis (CanL) in Mediterranean countries (either alone or in combination with allopurinol) [6]. However, despite their proven leishmanicidal activity, SbV-based drugs have some drawbacks, including high price, undesired side effects following parenteral administration and development of resistances, which encourage their replacement by safer treatments [7,8]. Likewise, allopurinol resistance has also recently been reported in *L. infantum* parasites isolated from dogs undergoing allopurinol treatment, and associated with clinical relapse [9]. Other oral therapies, such as miltefosine, have shown a high incidence of resistance as well as many adverse effects including teratogenicity and gastrointestinal disorders [10].

The natural susceptibility and clinical evolution of CanL patients strongly depends on the type of immune response elicited in the dog after infection. According to Reis et al. [11], the evaluation of different factors such as parasite burden, humoral response, cell-mediated immunity, or cytokine expression, have a predictive value on the progression of infection and should, therefore, be considered as key markers of resistance and susceptibility to CanL. Increased levels of certain parameters such as parasite load, IL-10, TGF-β, or *Leishmania*-specific IgG, IgM, IgA, and IgE serum antibodies are indicators of disease progression. By contrast, increased levels of peripheral blood mononuclear cells (PBMC) proliferation after leishmanial antigen stimulation, interferon gamma (IFN-γ) and tumor necrosis factor alpha (TNF-α) expression, proliferation of CD4+, CD8+, and B-cell subsets, or a positive *Leishmania* skin test (LST) are associated with resistance [12]. Accordingly, increased levels of IFN-γ in cured cases have been reported [13]. Previously, it has also been described that an elevation in the production of IL-10 is associated with deficient CD4+ T cell effector responses during clinical visceral leishmaniasis. In dogs, CD4+ and CD8+ T cell exhaustion has been described as a significant stepwise loss of Ag-specific proliferation and IFN-γ production, linked to clinical worsening [14].

Nucleotides are low molecular weight intracellular compounds that play key roles in biochemical processes. Sources include de novo synthesis, recovery via salvage mechanisms, and dietary intake. Although endogenous production serves as the main nucleotide source, evidence suggests that exogenous supply is essential to immune competence, intestinal development, and recovery. Thus, nucleotides become essential in certain situations where there is physiological stress and an increased demand for nucleic acid synthesis, including immunosuppression, infection, and some disease states [15]. Orally administered nucleotides modulate the immune response, having a positive impact on lipid metabolism and immunity, tissue growth, development, and repair. They can also be particularly beneficial in situations when rapidly proliferating tissues, such as the intestine and the immune system, fail to fulfil their nucleotides needs by de novo synthesis. Therefore, dietary nucleotides are considered as potential immuno-modulatory compounds [16,17].

On the other hand, AHCC^®^, a standardized extract of cultured *Lentinula edodes* mycelia (ECLM), stimulates the immune system in humans [18]. Among other actions, AHCC has been reported to increase Th1 cell responses [18,19,20,21] and to exert immunomodulatory effects on intestinal epithelial cells and macrophages [22], probably throughout the regulation of TLR2 and TLR4 signaling [23], which could potentially benefit *Leishmania-*infected dogs.

In a recent multicenter open-label positively-controlled clinical trial, sixty-nine dogs with naturally-occurring clinical leishmaniosis were randomized to receive allopurinol or nucleotides plus AHCC orally once daily for 180 days in addition to an initial 28-day course of methylglucamine antimoniate (MGA). The combined use of MGA and nucleotides plus AHCC resulted in improved clinical scores and an overall amelioration in the biomarkers used to monitor response to treatment, showing similar efficacy to MGA plus allopurinol, and without producing xanthinuria [24]. Furthermore, in another recent multicenter, randomized, double-blind, placebo-controlled trial, 1-year administration of dietary nucleotides plus AHCC in clinically healthy dogs naturally infected with *L. infantum* led to a significantly reduced disease progression rate compared to placebo, and a decrease in the levels of anti-*Leishmania* antibodies indicating that dietary nucleotides plus AHCC could potentially be used as a preventive approach [25].

The present work was aimed at assessing the in vitro effects of nucleotides and AHCC on murine lymphocyte proliferation and cytokine production upon stimulation with leishmanial antigens as well as on *Leishmania* infectivity to macrophages cocultured with lymphocytes to better characterize the cellular and molecular bases of the potential immunomodulatory effects of these compounds.

## 2. Results

### 2.1. Cytotoxicity Studies on Murine Macrophages

The toxicity of the nucleotides and AHCC alone was tested by preparing serial dilutions of both compounds starting at concentrations of 400 μg/mL. No cell toxicity was observed for any of the compounds at any of the tested dilutions, suggesting that the CC_50_ (cytotoxic concentration 50) was well above 400 μg/mL (see Supplementary Material 1).

### 2.2. Production of Nitric Oxide (NO) by Macrophages

The production of NO by macrophages was not altered following the application of compounds alone or with any of the combinations (see Supplementary Material 2) suggesting that the in vivo efficacy of nucleotides plus AHCC in CanL patients previously reported [24,25] probably occurs through a different mechanism of action.

### 2.3. In Vitro Promastigote and Amastigote Susceptibility Assays

No in vitro changes were observed with the compounds alone or with any of the combinations on parasites growth, thus indicating that nucleotides and AHCC do not elicit a direct activity against the parasites (see Supplementary Material 3).

### 2.4. In Vitro Lymphoproliferation Assay

**Figure 1:**
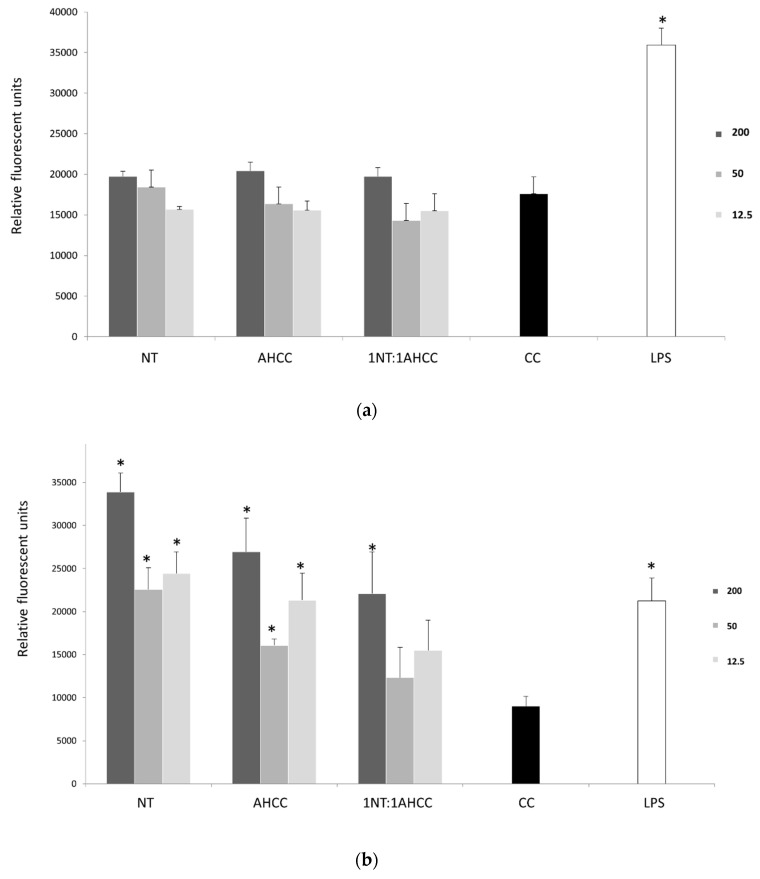
Effect of nucleotides (NTs), AHCC, and their 1:1 combination at concentrations of 200, 50, and 12.5 µg/mL on lymphocyte proliferation before (**a**) and after (**b**) soluble *Leishmania infantum* antigen (SLA) stimulation. * *p* < 0.05 versus control cells (CC).

Nucleotides and AHCC, tested alone or at a 1:1 ratio at final concentrations of 200, 50, and 12.5 µg/mL, did not show any significant (*p* < 0.05) proliferation enhancement effects on murine lymphocytes compared to the control (Figure 1a). In contrast, after soluble *Leishmania infantum* antigen (SLA) stimulation, nucleotides and the 1:1 combination of nucleotides with AHCC were able to significantly increase lymphocyte proliferation compared to the control (Figure 1b), hence counteracting the antiproliferative effect exerted by the parasite antigen.

### 2.5. Analysis of Cytokines

**Figure 2:**
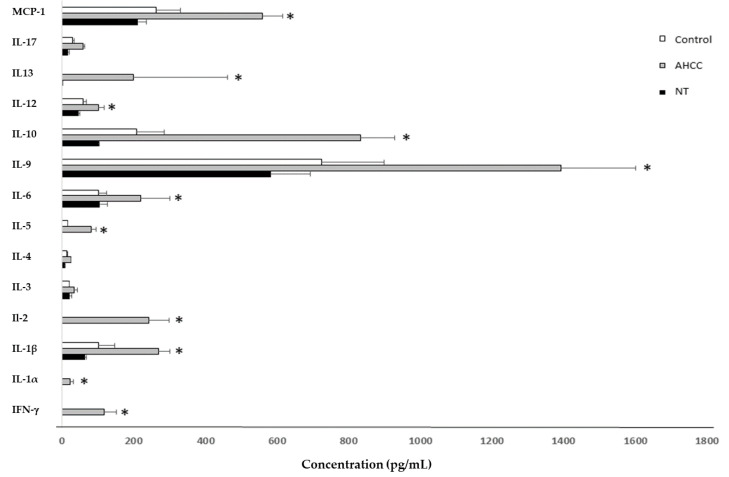
Comparison of cytokine production by lymphocytes isolated from BALB/c mouse spleen after 24 h exposure to nucleotides (NTs) and AHCC. * *p* < 0.05 versus the control.

A concentration of 200 μg/mL was used for the test compounds in the studies aimed at quantifying cytokine production because this was the one that provided the best results in the proliferation studies (Figure 1a,b). Moreover, given that the proliferation studies revealed that the addition of the compounds could counteracted the antiproliferative effect of SLA (Figure 1b), cytokine levels were quantified in naïve but also in *L. infantum*-infected macrophage/lymphocyte cocultures (Table 1 and Figure 2).

#### 2.5.1. Coculture of Lymphocytes and Naïve Macrophages

**Figure 3:**
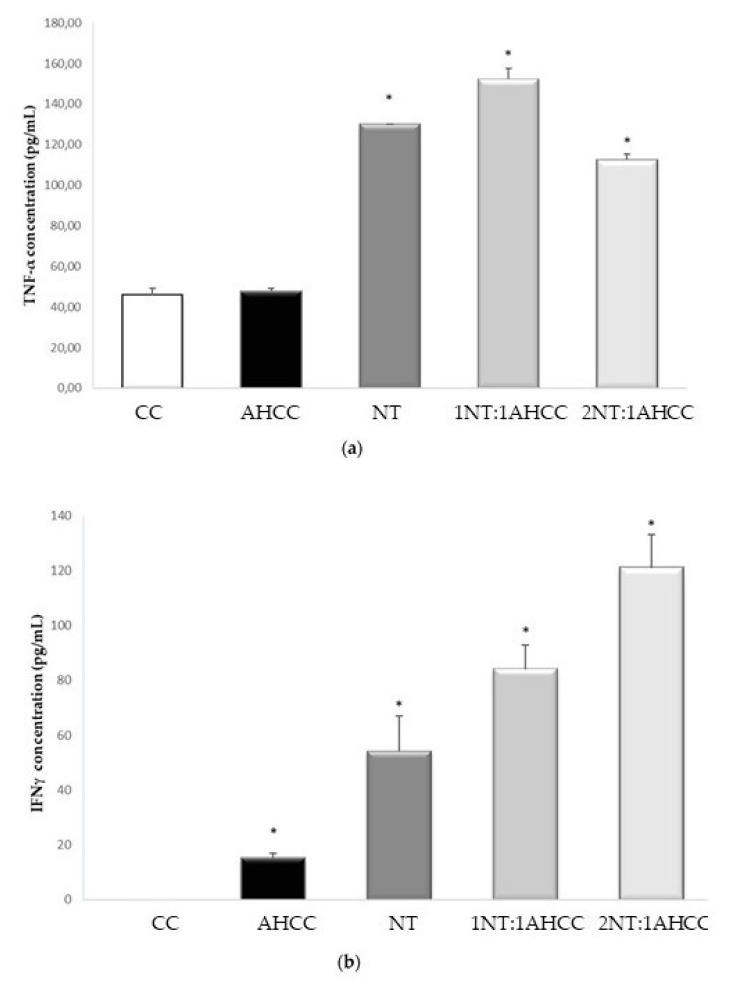
TNF-α (**a**) and IFN-γ (**b**) quantification in coculture of lymphocytes and naïve macrophages exposed to nucleotides (NTs) and AHCC, alone or in combination. * *p* < 0.05 versus the control.

The results are shown in Table 2. TNF-α levels were significantly increased with nucleotides either alone or in combination with AHCC, compared to the control (Figure 3a). Nucleotides, AHCC, and their 1:1 and 2:1 combinations led to significantly increased levels of IFN-γ, compared to the control (Figure 3b). Similar effects were found for IL-1β, IL-6, IL-12, IL-17, and RANTES. The combination of both compounds led to a significant increase in IL-9. The levels of MCP-1 were significantly increased by AHCC and the 1:1 and 2:1 combinations with nucleotides, compared to the control.

#### 2.5.2. Coculture of Lymphocytes and Leishmania-Infected Macrophages

**Figure 4:**
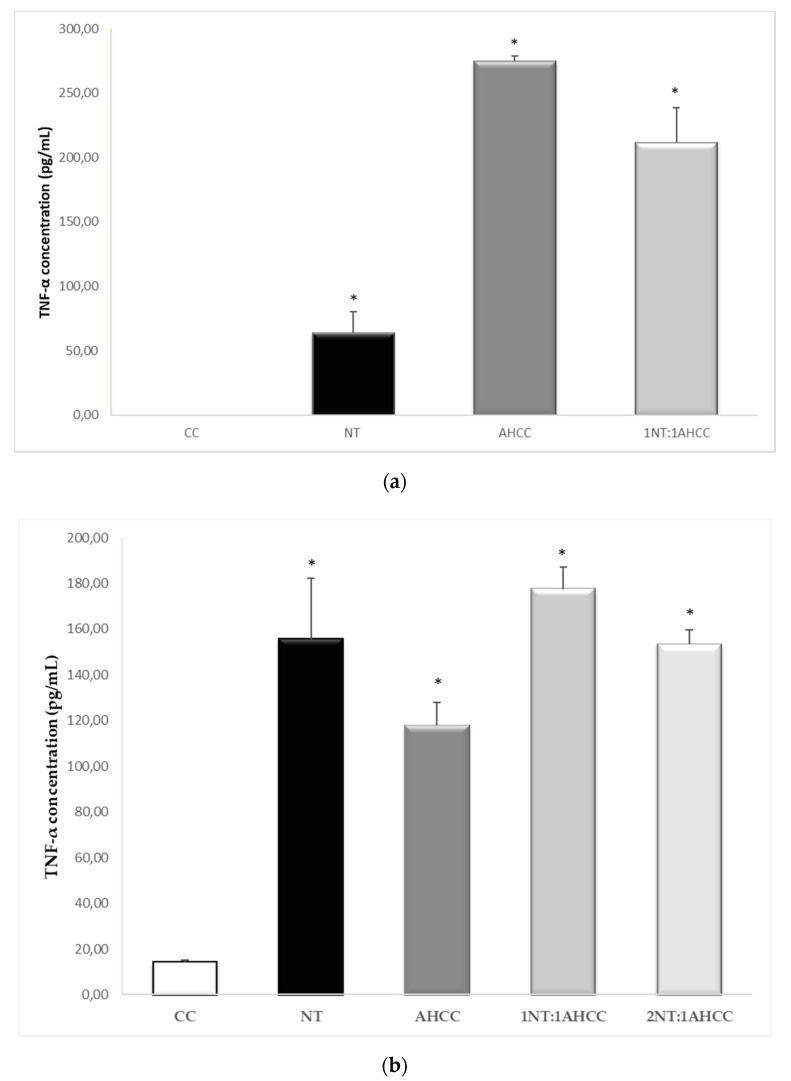
TNF-α determination after exposure to nucleotides (NTs) and AHCC, alone or in combination in *L*. *infantum*-infected macrophages (**a**) and *L. infantum*-infected macrophage/lymphocyte cocultures (**b**). * *p* < 0.05 versus the control.

**Figure 5:**
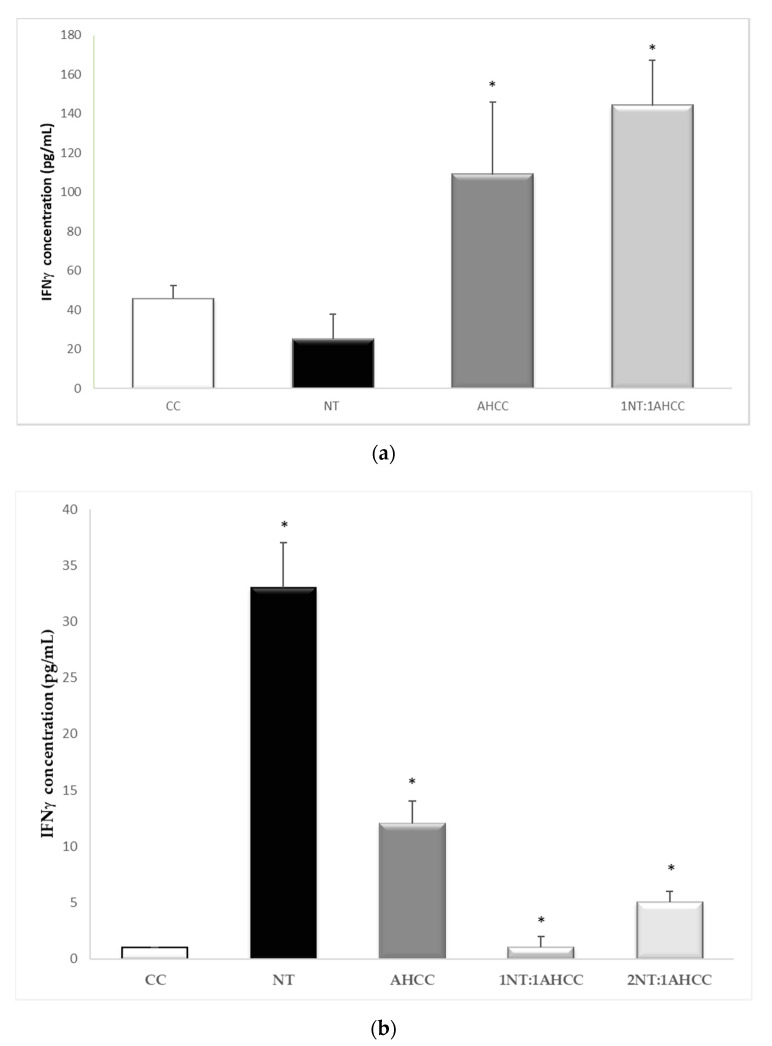
IFN-γ determination after exposure to nucleotides (NTs) and AHCC, alone or in combination, in *L*. *infantum*-infected macrophages (**a**) and *L. infantum*-infected macrophage/lymphocyte cocultures (**b**). * *p* < 0.05 versus the control.

A different cytokine stimulation profile was observed in the infected cocultures compared to the noninfected ones (Table 3). TNF-α production was significantly (*p* < 0.05) enhanced by nucleotides, AHCC, and their combinations (Figure 4). IFN-γ was increased in the presence of nucleotides, AHCC, and their 2:1 combination (Figure 5). The levels of IL-1α were also increased by the presence of nucleotides and AHCC, alone and in combination. By contrast, the production of IL-1β was not significantly stimulated in the infected coculture. The levels of IL-6 were enhanced significantly only in the presence of AHCC.

**Figure 6:**
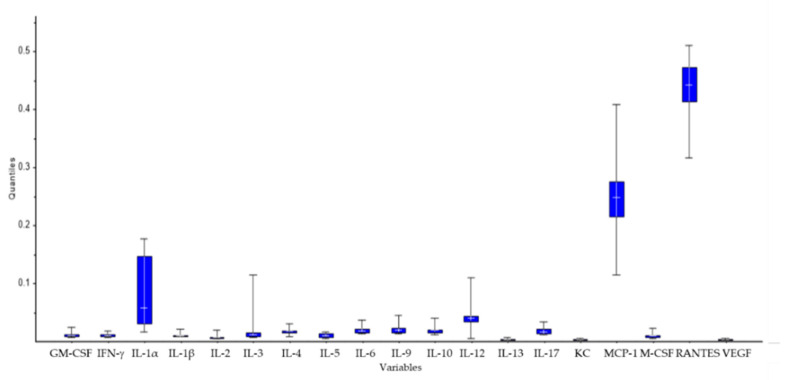
Multivariate analysis on the cytokine profile obtained in *Leishmania*-infected cocultures of lymphocytes and macrophages in the presence of nucleotides and AHCC.

MCP-1 and RANTES levels were significantly increased with both compounds, alone or in combination. MVA analysis of all cytokine levels showed that most of the variability of the cytokine profile among the different groups was due to the levels of IL-1α, MCP-1, and RANTES (Figure 6).

## 3. Discussion

*Leishmania infantum* is often considered an opportunistic parasite which benefits from an inadequate immune response, especially in children or immunosuppressed human patients. It is well known that the type of immune response is crucial for the progression and control of the disease. The activation of infected macrophages is required to kill the intracellular *Leishmania* parasites responsible for the disease [26]. An effective cellular immune response (Th1) is characterized by the release of pro-inflammatory cytokines such as IL-2, IFN-γ, and TNF-α, while the predominance of a Th2 profile is marked by the overexpression of TGF-β and IL-10, and is associated with a poorer prognosis [11].

Thereby, immunocompromised patients might benefit from natural compounds that could modulate the immune response towards a Th1 profile. In the in vitro studies reported herein, we have shown that both nucleotides and AHCC are not cytotoxic even at high concentrations, that they enhance lymphocyte proliferation after SLA stimulation, and that they possess the ability of modulating the immune response both in healthy macrophage/lymphocyte cocultures as well as in *Leishmania*-infected macrophage/lymphocyte cocultures. Our data reveal that there is a synergistic effect between both compounds, and that their 1:1 weight ratio combination leads to the best results. This positive impact on the immune system and enhancement of the Th1 response could explain the beneficial effects previously reported in vivo after the oral administration of nucleotides and AHCC in CanL patients using a similar ratio [24,25]. However, further studies evaluating the specific effects of such compounds on Th1 cells would be warranted.

It is worthy to mention that, according to the results reported herein, none of the tested compounds possess a direct anti-*Leishmania* activity per se and that they do not promote the NO production by macrophages. The enhanced immune response elicited by nucleotides and AHCC may be related to the IFN-γ activation of macrophages leading to TNF-α production, which, in turn, might increase the levels of reactive oxygen species (ROS) in PBMC and, subsequently, result in *Leishmania* destruction [27].

Prior in vivo benefits observed in dogs could be explained by a combination of factors, such as: stimulation of TNF-α, IFN-γ, MCP-1, RANTES, and IL-1α; decreased IL-6 and IL-9 levels. Previously, it has been reported that nucleotides play a key role as regulators of immune functions able to increase the Th1/Th2 ratio [17,28]. AHCC has also been shown to exert a protective effect against pathogens by enhancing CD4 and CD8 T cell immune responses via increasing the production of TNF-α and IFN-γ. This protective effect was maintained even 30 days after discontinuing AHCC oral administration [20]. Moreover, it has been shown that AHCC also promotes T helper (Th17) and Th1 cell responses via inducing IL-1β production from monocytes in humans [18]. Surprisingly, this effect was observed in our studies in the naïve cocultures but not in the infected cocultures.

Furthermore, the modulation of the immune system by nucleotides and AHCC appears to be more complex and possibly not only related to the stimulation of IFN-γ and TNF-α. This is the first report that shows that nucleotides and AHCC elicit a positive effect on MCP-1 and RANTES. The presence of MCP-1 is crucial in regulating Th1/Th2 balance in experimental leishmaniasis and it has been identified as a marker of cure of leishmaniasis in humans [29]. Moreover, in lesions of cutaneous leishmaniasis, a synergistic action has been described between MCP-1 and IFN-γ stimulating the killing of parasites by activating monocytes to clear intracellular parasites and promote healing [30]. On the other hand, RANTES also acts as a chemokine able to recruit monocytes/macrophages and eosinophils to the infection site hence being beneficial for the eradication of the parasites [31]. In our studies, nucleotides and AHCC showed the ability to decrease the levels of both IL-6 and IL-9 in infected macrophage/lymphocyte cocultures, which would benefit leishmaniasis patients. IL-9 is a susceptibility factor in *Leishmania* infection by promoting detrimental Th2 responses [32], and elevated levels of IL-6 have been correlated with a high severity of visceral leishmaniasis [33].

The data reported herein, together with previously published clinical data on the use of nucleotides plus AHCC [24,25], further support the interest of these products for their potential use in the management of canine and human leishmaniasis. The control of such disease is complex due to the heterogeneity of the different species and genotypes and the reported resistance to the most commonly used drugs [34]. Furthermore, this intervention could serve as complementary tool as part of a One Health multimodal approach together with other immunotherapy options [35]. In fact, recent publications include nucleotides and AHCC within the choices of effective immunotherapies for the management and control of CanL [36,37].

## 4. Materials and Methods

### 4.1. Compounds

The compounds used in the study were nucleotides (Nucleoforce^®^ Dogs, Bioiberica S.A.U., Esplugues de Llobregat, Spain) and AHCC^®^ (ECLM, Amino Up Chemical Co. Ltd., Sapporo, Japan).

### 4.2. Mice

BALB/c mice of 6–8 weeks of age were purchased from Harlan Ibérica S.A. (Barcelona, Spain) and allocated in the Animal House Unit of the Complutense University under controlled feeding, light/darkness cycles, and temperature conditions. Animal handling was carried out according to the Principles and Guidelines for the Use of Animals in Research.

### 4.3. Cytotoxicity Studies on Murine Macrophages

The cytotoxicity assay was carried out as previously described [38]. J774 murine macrophages were grown in RPMI 1640 medium supplemented with 10% heat-inactivated FBS (30 min at 56 °C), penicillin G (100 U/mL) and streptomycin (100 μg/mL) [25]. For the experiments, cells in the preconfluence phase were harvested with 0.03% EDTA-0.05% trypsin in PBS for 20 min. Cell cultures were maintained at 37 °C in a humidified environment with 5% CO_2_ (Hucoa Erlöss, SA/Thermo Fisher Scientific, Madrid, Spain). For evaluation assays, J774 macrophages cell lines were seeded (5 × 104 cells/well) in 96-well flat-bottom microplates with 100 μL RPMI 1640 medium. The cells were allowed to attach for 24 h at 37 °C, 5% CO_2_, and the medium was replaced by different concentrations of the compounds resulting in a 200 μL total volume with a final concentration identical to the one described above. Macrophages were exposed to the compounds for 24 h. Growth controls were also included. Afterwards, a volume of 20 μL of the 2.5 mM resazurin solution was added, and plates were incubated for another 3 h to evaluate cell viability. The reduction in resazurin was determined by the fluorescence intensity (535 nm excitation wavelength and 590 nm emission wavelength). Relative Fluorescence Units (RFU) (535–590 nm excitation–emission wavelength) were determined with a fluorimeter (Infinite 200, Tecani-Control, Männedorf, Switzerland). Growth inhibition (%) was calculated using Equation (1):

(1) $$\mathrm{Growth}\;\mathrm{inhibition}\;\left(\%\right)=100-\frac{\mathrm{RFU}\;\mathrm{treated}\;\mathrm{wells}\;–\;\mathrm{RFU}\;\mathrm{signal}\;\mathrm{to}\;\mathrm{noise}}{\mathrm{RFU}\;\mathrm{untreated}\;–\;\mathrm{RFU}\;\mathrm{signal}\;\mathrm{to}\;\mathrm{noise}}\times 100$$

Each concentration was assayed in triplicate—400 µg/mL being the highest concentration tested. The medium and drug controls were used in each test as blanks, and the background was subtracted. The CC_50_ was calculated by probit analysis using SPSS 17.0 Statistics Software.

### 4.4. Quantification of NO Production by Macrophages

Quantification of NO production by macrophages was determined in the supernatants of spleen lymphocytes cultured together with lipopolysaccharide (LPS) or the test compounds (nucleotides, AHCC and their combination) for 24 h by using a Griess test [39]. An aliquot of 100 µL was incubated with 100 µL Griess reagent (1% sulfanilamide, 0.1% N-(1-naphthyl)ethylenediamine dihydrochloride in 2.5% ortho-phosphoric acid) for 5 min in darkness. Absorbance was measured at 570 nm.

### 4.5. In Vitro Promastigote and Amastigote Susceptibility Assays

In vitro activity studies were performed using *L. braziliensis, L. amazonensis, L. infantum*, and *L. donovani* as previously described [40,41]. Nucleotides and AHCC, alone or in combination (1:1, 2:1, 3:1, 1:3, and 1:2 w:w ratio), were diluted in culture media and tested at different concentrations starting at 200 μg/mL. Briefly, against extracellular promastigotes, 2.5 × 10^6^ parasites/well were seeded in 96-well microliter plates. The compounds were dissolved in DMSO and diluted in the culture medium at concentrations ranging from 100 to 0.8 μg/mL in a final volume of 200 μL. After incubation for 48 h at 26 °C, 20 μL of the 2.5 mM resazurin solution was added to each well and incubated for 3 h. The fluorescence intensity (535 nm excitation wavelength and 590 nm emission wavelength) was measured in a fluorometer (Infinite 200, Tecan Group Ltd., Männedorf, Switzerland). All assays were carried out in triplicate. Against intracellular amastigotes, 5 × 10^4^ macrophages and stationary promastigotes were seeded in a 1:10 ratio in each well of a microtiter plate, suspended in 200 μL culture medium and incubated for 24 h at 33 °C, 5% CO_2_ in a humidity chamber. After this first incubation, temperature was increased up to 37 °C for another 24 h. Thereafter, cells were washed several times in culture medium by centrifugation at 125× *g* for 5 min in order to remove free noninfective promastigotes. Finally, the supernatant was replaced by 200 μL/well culture medium containing compounds to be tested followed by incubation for 48 h. Then, the supernatants were collected and stored at −80 °C for cytokine quantification. After incubation of the plates for 48 h at 37 °C in 5% CO_2_, the culture medium was replaced with an equal volume of lysis solution (Schneider’s with 0.048% HEPES and 0.01% SDS) and maintained at room temperature for 20 min. The lysis solution was then replaced with Schneider’s medium followed by incubation at 26 °C for another 3 days to allow the transformation of viable amastigotes into promastigotes and their subsequent proliferation. Aliquots of 20 μL of 2.5 mM resazurin were added to each well, and the plates were incubated for 3 h. Finally, fluorescence emission was measured as described above. All assays were carried out in triplicate. The half maximal inhibitory concentration (IC_50_) was determined by a probit analysis.

### 4.6. Preparation of Leishmania Infantum Antigen

Soluble *Leishmania infantum* antigens (SLAs) were prepared from stationary promastigotes of M/CAN/ES/96/BCN150 isolate of *L. infantum* cultured in Schneider medium to which HEPES (4 g/L), sodium bicarbonate (0.4 g/L), 10% SBF, and antibiotics were added. After washing several times in PBS pH 7.2–7.4, the parasite suspension, kept on ice, was sonicated until a visible decrease in viscosity was achieved. Subsequently, overnight extraction was performed at 4 °C in 0.5 M Tris-HCl buffer pH 7.5 followed by centrifugation at 13,200× *g* for 1 h, and then, the supernatant was collected. Protein content was estimated by the Bradford assay.

### 4.7. In Vitro Lymphoproliferation Assay

One BALB/c mouse spleen was homogenized through a stainless-steel tissue grinder in RPMI medium supplemented with 2 mmol/L L-glutamine, 10 mmol/L Hepes, sodium bicarbonate (2.2 g/L), and gentamicin (50 μg/mL). The cell suspension was treated with NH4Cl at 0.8% to remove red cells, and thereafter, cells were washed three times in RPMI by centrifugation at 125× *g* for 5 min. After washing, cells were counted using the Newbauer hemocytometer and their viability was determined by Trypan blue staining exclusion. Cell suspensions were adjusted to a concentration of 5 × 10^5^ cell/mL and then dispensed to 96-well microtiter plates (Sarstedt, Barcelona, Spain). LPS (Sigma, Madrid, Spain) and SLAs were used at concentrations of 2.5 and 20 μg/mL, respectively. Plates were incubated a 37 °C, 5% CO_2_ for 3 days. Following incubation, a volume of 20 μL of the 2.5 mM resazurin solution in PBS was added, and the plates were incubated for 3 h under the same conditions. The reduction in resazurin was determined as described above.

### 4.8. Coculture of Macrophages and Lymphocytes

In this study, we adapted a method from Viana et al., 2013 [42]. Briefly, lymphocytes from BALB/c spleen and J774 macrophages were used. Two different coculture systems were tested: lymphocyte/naïve macrophages and lymphocyte/*L. infantum*-infected macrophages. A 1:1 ratio was used in both systems. Both culture systems were incubated for 48 h with nucleotides and AHCC, and after this period, the supernatants were collected and stored at −80 °C for cytokines quantification.

### 4.9. Analysis of Cytokines

The level of cytokines was determined in the supernatants of spleen lymphocytes cultured with LPS or compounds (nucleotides, AHCC or both). After incubation for 24 h, the supernatants were collected and stored at −80 °C for cytokine quantification. The analyses were carried out by using the Quantibody^®^ array (RayBiotech, Madrid, Spain), a multiplexed sandwich ELISA-based quantitative array platform. Nineteen different markers were analyzed: granulocyte-macrophage colony-stimulating factor (GM-CSF); IFN-γ; interleukins (IL) IL-1α, IL-1β, IL-2, IL-3, IL-4, IL-5, IL-6, IL-9, IL-10, IL-12, IL-13, and IL-17; growth-regulated alpha protein (KC); monocyte chemoattractant protein-1 (MCP-1); macrophage colony-stimulating factor (M-CSF), regulated on activation; normal T cell expressed and secreted (RANTES); and vascular endothelial growth factor (VEGF). A pair of cytokine specific antibodies was used for detection. A capture antibody was first bound to the glass surface. After incubation with the sample, the target cytokine was trapped on the solid surface. A second biotin-labeled detection antibody was added, which recognized a different epitope of the target cytokine. The cytokine–antibody–biotin complex was visualized through the addition of the streptavidin-conjugated Cy3 equivalent dye, using a laser scanner (GenePix^®^ 4000B Scanner, UCM facilities).

A sandwich ELISA kit was used to determine the concentrations of TNF-α. The procedure was carried out according the manufacturer’s instructions. Briefly, standards and samples (50 µL) were diluted with 50 µL commercial diluting solution in triplicate and incubated for 2 h at 37 °C. Plates were washed five times and 100 µL conjugated anti-TNF-α was added and incubated for 1 h at 37 °C. Finally, 100 µL TMB was added as substrate and incubated for 30 min in the dark at room temperature. The reaction was stopped with 3N H_2_SO_4_, and the optical densities (OD) were read at 450 nm.

### 4.10. Statistical Data Analysis

Lymphoproliferation results were analyzed by a Mann–Whitney U test. A multivariate data analysis was performed using The Unscrambler^®^ X software (CAMO Software, Oslo, Norway). The cytokine profile of infected macrophage/lymphocyte cocultures was analyzed by Principal Component Analysis (PCA) in order to the study systematic variability and the relationships between variables and scores (presence of nucleotides and AHCC and their combinations). A Singular Value Decomposition algorithm was employed. The correlation loadings of the PCAs were represented to understand the variance for each variable for a given PCA, giving information about the source of the variability inside the dataset.

## 5. Conclusions

Nucleotides and AHCC showed the ability to enhance an effective Th1 immune response in vitro by increasing TNF-α, IFN-γ, MCP-1, RANTES, and IL-1α levels while reducing IL-6 and IL-9 cytokine levels in *Leishmania*-stimulated/infected murine immune cells. Since a stronger cellular immune response in leishmaniasis is associated with a better prognosis, the combination of nucleotides plus AHCC could be beneficial for the prevention and management of CanL, and it might also be helpful in human visceral leishmaniasis.

## Figures and Tables

**Table 1:** Cytokine quantification after exposure to nucleotides (NTs), AHCC, and their combinations in lymphocyte culture with or without SLA stimulation. Each value represents the mean +/− standard deviation for three replicates. * *p* < 0.05 versus the control.

Concentration (pg/mL)	CC	NT	1NT:1AHCC	2NT:1AHCC	NT + SLA	1NT:1AHCC + SLA	2NT:1AHCC + SLA	LPS
GM-CSF	0 +/− 0	0 +/− 0	0 +/− 0	1 +/− 0	2 +/− 0	1 +/− 0	2 +/− 0	0 +/− 0
IFN-γ	3 +/− 0	2 +/− 0	4 +/− 0	2 +/− 0	9 +/− 0 *	5 +/− 0 *	9 +/− 0 *	4 +/− 0
IL-1α	0 +/− 0	0 +/− 0	0 +/− 0	0 +/− 0	0 +/− 0	0 +/− 0	0 +/− 0	2 +/− 1
IL-1β	1 +/− 0	3 +/− 1	1 +/− 0	1 +/− 0	3 +/− 0	1 +/− 0	3 +/− 0	0 +/− 0
IL-2	0 +/− 0	2 +/− 0	1 +/− 0	7 +/− 1 *	9 +/− 1 *	8 +/− 0 *	11 +/− 1 *	0 +/− 0
IL-3	0 +/− 0	0 +/− 0	0 +/− 0	0 +/− 0	0 +/− 0	0 +/− 0	0 +/− 0	0 +/− 0
IL-4	0 +/− 0	0 +/− 0	0 +/− 0	0 +/− 0	0 +/− 0	0 +/− 0	0 +/− 0	0 +/− 0
IL-5	0 +/− 0	0 +/− 0	0 +/− 0	0 +/− 0	0 +/− 0	0 +/− 0	0 +/− 0	0 +/− 0
IL-6	4 +/− 0	5 +/− 0	19 +/− 2 *	24 +/− 2 *	9 +/− 1 *	25 +/− 3 *	26 +/− 2 *	1229 +/− 7 *
IL-9	48 +/− 5	62 +/− 10	50 +/− 4	79 +/− 3 *	103 +/− 10 *	33 +/− 3	114 +/− 7 *	138 +/− 12 *
IL-10	0 +/− 0	0 +/− 0	5 +/− 0	19 +/− 1 *	5 +/− 0	0 +/− 0	0 +/− 0	45 +/− 2 *
IL-12	2 +/− 0	2 +/− 0	4 +/− 0	5 +/− 0 *	5 +/− 0 *	1 +/− 0	4 +/− 0	4 +/− 0
IL-13	0 +/− 0	0 +/− 0	0 +/− 0	0 +/− 0	0 +/− 0	0 +/− 0	0 +/− 0	0 +/− 0
IL-17	0 +/− 0	0 +/− 0	0 +/− 0	0 +/− 0	1 +/− 0	0 +/− 0	0 +/− 0	1 +/− 0
KC	0 +/− 0	0 +/− 0	0 +/− 0	0 +/− 0	0 +/− 0	0 +/− 0	0 +/− 0	38 +/− 1
MCP-1	0 +/− 0	12 +/− 1 *	4 +/− 0	8 +/− 1 *	12 +/− 1 *	0 +/− 0	9 +/− 0 *	0 +/− 0
M-CSF	0 +/− 0	0 +/− 0	0 +/− 0	0 +/− 0	0 +/− 0	0 +/− 0	1 +/− 0	0 +/− 0
RANTES	1 +/− 0	1 +/− 0	0 +/− 0	1 +/− 0	0 +/− 0	0 +/− 0	0 +/− 0	82 +/− 2 *
TNF-α	0 +/− 0	0 +/− 0	0 +/− 0	0 +/− 0	12 +/− 1 *	0 +/− 0	10 +/− 0 *	0 +/− 0
VEGF	0 +/− 0	0 +/− 0	0 +/− 0	0 +/− 0	0 +/− 0	0 +/− 0	0 +/− 0	1 +/− 0

**Table 2:** Cytokine quantification after exposure to nucleotides (NTs), AHCC, and their combinations in cocultures of lymphocytes and naïve macrophages. Each value represents the mean +/− standard deviation for three replicates. * *p* < 0.05 versus the control.

Concentration (pg/mL)	CC	AHCC	NT	1NT:1AHCC	2NT:1AHCC
GM-CSF	0 +/− 0	0 +/− 0	0 +/− 0	0 +/− 0	0 +/− 0
IFN-γ	0 +/− 0	15 +/− 2 *	54 +/− 13 *	84 +/− 9 *	121 +/− 12 *
IL-1α	0 +/− 0	24 +/− 12 *	103 +/− 19 *	36 +/− 10 *	26 +/− 11 *
IL-1β	0 +/− 0	0 +/− 0	20 +/− 5 *	84 +/− 3 *	113 +/− 12 *
IL-2	0 +/− 0	0 +/− 0	1 +/− 0	−	1 +/− 0
IL-3	0 +/− 0	0 +/− 0	0 +/− 0	6 +/− 1 *	6 +/− 1 *
IL-4	0 +/− 0	0 +/− 0	0 +/− 0	0 +/− 0	0 +/− 0
IL-5	0 +/− 0	0 +/− 0	2 +/− 1	26 +/− 7 *	32 +/− 10 *
IL-6	50 +/− 6	86 +/− 9 *	60 +/− 5	168 +/− 18 *	133 +/− 8 *
IL-9	188 +/− 29	0 +/− 0	134 +/− 21	341 +/− 47 *	382 +/− 64 *
IL-10	0 +/− 0	0 +/− 0	0 +/− 0	0 +/− 0	18 +/− 3 *
IL-12	4 +/− 1	1 +/− 0	14 +/− 1 *	22 +/− 3 *	28 +/− 2 *
IL-13	0 +/− 0	0 +/− 0	0 +/− 0	0 +/− 0	0 +/− 0
IL-17	5 +/− 1	4 +/− 1	0 +/− 0	22 +/− 4 *	18 +/− 2
TNF-α	45.7+/− 3.5	47.72+/−1.5	129.94+/− 0 *	152.1+/− 5.4 *	112.44+/− 2.74 *
KC	0 +/− 0	0 +/− 0	0 +/− 0	0 +/− 0	0 +/− 0
MCP-1	604 +/− 64	1133 +/− 303 *	715 +/− 159	830 +/− 105 *	941 +/− 143 *
M-CSF	0 +/− 0	0 +/− 0	0 +/− 0	0 +/− 0	0 +/− 0
RANTES	191 +/− 14	194 +/− 22	181 +/− 8	234 +/− 17 *	261 +/− 10 *
VEGF	2 +/− 1	0 +/− 0	0 +/− 0	2 +/− 0	3 +/− 0

**Table 3:** Cytokine quantification after exposure of lymphocytes and *L. infantum*-infected macrophages to nucleotides (NTs), AHCC, and their combinations. Each value represents the mean +/− standard deviation for three replicates. * *p* < 0.05 versus the control.

Concentration (pg/mL)	Noninfected Coculture	Coculture	AHCC	NT	1NT:1AHCC	2NT:1AHCC
GM-CSF	0 +/− 0	0 +/− 0	0 +/− 0	0 +/− 0	0 +/− 0	0 +/− 0
IFN-γ	0 +/− 0	1 +/− 0	12 +/− 2 *	33 +/− 4 *	1 +/− 0	5 +/− 1 *
IL-1α	0 +/− 0	0 +/− 0	85 +/− 83 *	97 +/− 80 *	29 +/− 12 *	122 +/− 51 *
IL-1β	0 +/− 0	0 +/− 0	0 +/− 0	0 +/− 0	0 +/− 0	0 +/− 0
IL-2	0 +/− 0	12 +/− 1 *	0 +/− 0	0 +/− 0	0 +/− 0	0 +/− 0
IL-3	0 +/− 0	0 +/− 0	0 +/− 0	0 +/− 0	0 +/− 0	0 +/− 0
IL-4	0 +/− 0	0 +/− 0	0 +/− 0	0 +/− 0	0 +/− 0	0 +/− 0
IL-5	0 +/− 0	0 +/− 0	0 +/− 0	0 +/− 0	3 +/− 1	0 +/− 0
IL-6	50 +/− 6	34 +/− 4	91 +/− 19 *	43 +/− 2	57 +/− 3	44 +/− 7
IL-9	188 +/− 29	223 +/− 37	0 +/− 0	33 +/− 3 *	161 +/− 20	53 +/− 13 *
IL-10	0 +/− 0	2 +/− 0	0 +/− 0	10 +/− 0 *	26 +/− 1 *	0 +/− 0
IL-12	4 +/− 1	0 +/− 0	1 +/− 0	8 +/− 1	13 +/− 1 *	3 +/− 1
IL-13	0 +/− 0	0 +/− 0	0 +/− 0	0 +/− 0	0 +/− 0	0 +/− 0
IL-17	5 +/− 1	0 +/− 0	0 +/− 0	0 +/− 0	1 +/− 0	0 +/− 0
TNF-α	45.7+/− 3.5	14.11+/−1.18	117.72+/−10.21 *	155.50+/−26.71 *	177.44+/−9.82 *	153.28+/−6.28 *
KC	0 +/− 0	0 +/− 0	0 +/− 0	0 +/− 0	0 +/− 0	0 +/− 0
MCP-1	604 +/− 64	764 +/− 100	2410 +/− 327 *	2144 +/− 269 *	2096 +/− 540 *	1318 +/− 546 *
M-CSF	0 +/− 0	0 +/− 0	0 +/− 0	12 +/− 2	0 +/− 0	0 +/− 0
RANTES	191 +/− 14	149 +/− 22	347 +/− 45 *	304 +/− 19 *	308 +/− 14 *	319 +/− 22 *
VEGF	2 +/− 1	0 +/− 0	2 +/− 0	6 +/− 1	5 +/− 1	2 +/− 0

## Supplementary Materials

The following are available online, S1: Cytotoxicity studies on murine macrophages. S2: Production of NO by macrophages. S3: In vitro promastigote and amastigote susceptibility assays.
