# Supplementary material for: Nucleotides and AHCC Enhance Th1 Responses In Vitro in Leishmania-Stimulated/Infected Murine Cells
Source: Molecules. 2020 Aug 27;25(17):3918. doi: 10.3390/molecules25173918 (PMC7504588; doi:10.3390/molecules25173918)
Supplement: Supplementary file 1 [file molecules-25-03918-s001.pdf]

Supplemental Material

Nucleotides and AHCC Enhance Th1 Responses In Vitro in *Leishmania*-Stimulated/Infected Murine Cells

M. Auxiliadora Dea-Ayuela [1], Sergi Segarra [2,*], Dolores R. Serrano [3,4] and Francisco Bolás-Fernández [5]

[1] Departamento de Farmacia. Facultad de Ciencias de la Salud, Universidad CEU-Cardenal Herrera, 46113 Moncada, Spain; mda_3000@yahoo.es
[2] R & D Bioiberica S.A.U., Av. dels Països Catalans 34, 08950 Esplugues de Llobregat, Spain
[3] Department of Pharmaceutics and Food Technology, School of Pharmacy, Universidad Complutense de Madrid, Plaza Ramon y Cajal s/n, 28040 Madrid, Spain; dr.serrano@farm.ucm.es
[4] Instituto Universitario de Farmacia Industrial (IUFI), School of Pharmacy, Universidad Complutense de Madrid, Avenida Complutense, 28040 Madrid, Spain
[5] Departament of Microbiology and Parasitology, School of Pharmacy, Universidad Complutense de Madrid, Plaza Ramon y Cajal s/n, 28040 Madrid, Spain; francisb@farm.ucm.es
* Correspondence: ssegarra@bioiberica.com; Tel.: +34-934904908

Table of Contents

SUPPLEMENTARY MATERIAL 1 - Cytotoxicity Studies on Murine Macrophages

Supplementary Material 1 - Cytotoxicity Studies on Murine Macrophages 3
Table 1. Percentages of Inhibition and CC_{50} in J774 Macrophages with The Reference Drug Miltefosine 3
Table 2. Percentages of Inhibition and CC50 In J774 Macrophages with Different Concentrations of Nucleotides (NT) and AHCC 4

Supplementary Material 2 - Production of No by Macrophages 5

Figure 1. Production of NO By Macrophages 5

Supplementary Material 3 - In Vitro Promastigote and Amastigote Susceptibility Assays 6

Table 1. Percentage of Growth Inhibition of Different Leishmania Species with Reference Drug Miltefosine**.** 6
Table 2. Percentage of Growth Inhibition of Leishmania Amazonensis with Nucleotides (NT) and AHCC. 7
Table 3. Percentage of Growth Inhibition of Leishmania Braziliensis with Nucleotides (NT) And AHCC. 7

Table 4. Percentage of Growth Inhibition of Leishmania Donovani with Nucleotides (NT) And AHCC. 8
Table 5. Percentage of Growth Inhibition of Leishmania Infantum with Nucleotides (NT) And AHCC. 8
Table 6. Percentage of Growth Inhibition of Different Leishmania Species with Reference Drug Miltefosine. 9
Table 7. Percentage of Growth Inhibition of Leishmania Infantum with Nucleotides (NT) And AHCC. 9
Table 8. Percentage of Growth Inhibition of Leishmania Amazonensis with Nucleotides (NT) 10

Supplementary Material 1

Cytotoxicity studies on murine macrophages

Growth inhibition (%) of macrophages was calculated by 100 - [(RFU treated wells – RFU signal-to-noise)/(RFU untreated – RFU signal-to-noise) x 100]. Cytotoxicity effect of compounds was defined as the 50% reduction of cell viability of treated culture cells with respect to untreated culture (CC_{50}).

Table 1. Percentages of inhibition and CC_{50} in J774 macrophages with the reference drug miltefosine.

**Concentration (μg/mL)**	***% inhibition***	
100.00	99	±0
50.00	27	±7
25.00	3	±2
12.50	2	±1
6.25	9	±1
3.13	6	±1
1.56	0	±2
0.78	-5	±0
CC_{50}	55.49±1.1	

Table 2. Percentages of inhibition and CC_{50} in J774 macrophages with different concentrations of nucleotides (NT) and AHCC.

Concentration (µg/mL)	AHCC		NT	
**400**	-2.48	±0.53	0.42	±2.28
**200**	-0.32	±3.60	0.27	±3.83
**100**	1.41	±7.19	0.12	±3.80
**50**	-0.55	±2.46	-2.25	±1.07
**25**	3.17	±2.94	3.82	±2.94
**12.5**	0.29	±3.37	-3.12	±7.22
**6.25**	-0.09	±4.32	2.06	±4.83
**3.12**	0.69	±4.89	-3.05	±3.60
**1.56**	-17.08	±3.46	4.59	±2.64
**0.78**	-0.94	±4.27	3.71	±1.28
**0.39**	3.23	±3.77	2.46	±2.34
**0.19**	-1.17	±1.87	-0.92	±1.08

Supplementary Material 2

Production of NO by macrophages

Quantification of NO production by macrophages was determined in the supernatants of spleen lymphocytes cultured together with LPS or compounds (nucleotides (NT), AHCC and their combination) for 24 h by using Griess test.

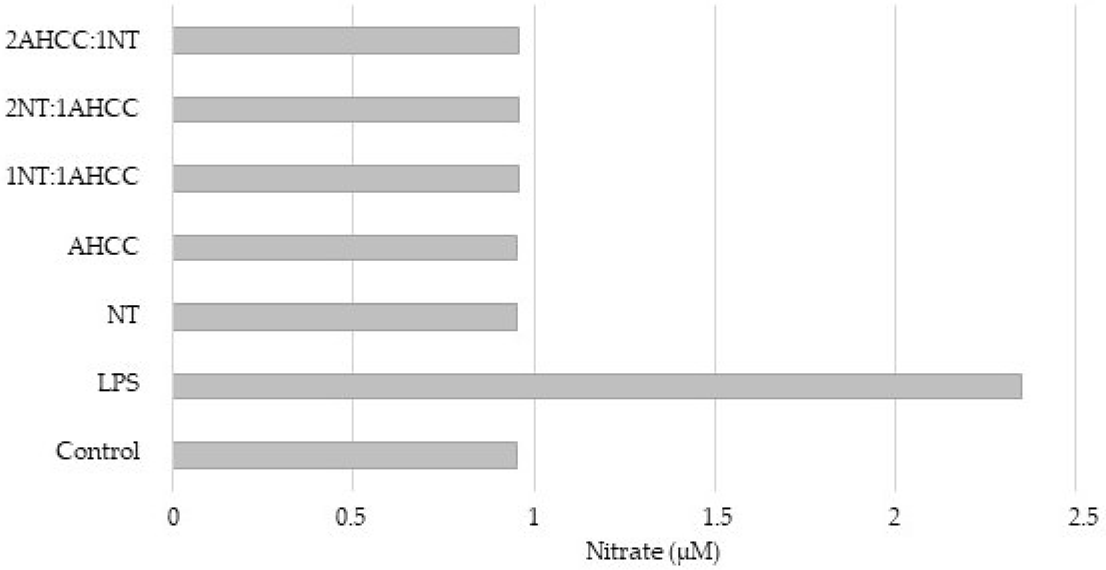

Figure 1. Production of NO by macrophages.

Supplementary Material 3

In vitro promastigote susceptibility assays

Growth inhibition (%) was calculated by 100 - [(RFU treated wells – RFU signal-to-noise)/(RFU untreated – RFU signal-to-noise) x 100]. Miltefosine was used as reference drug and was evaluated under the same conditions. The efficacy of each compound was estimated by calculating the IC_{50} (concentration of the compound that produced a 50% reduction in parasites) using a multinomial probit analysis

Table 1. Percentage of growth inhibition of different Leishmania species with reference drug miltefosine.

**Concentration (µg/mL)**	*L. amazonensis*		*L. infantum*		*L. braziliensis*		*L. donovani*	
100.00	93.4	±0.4	98.0	±0.4	94.6	±1.6	99.00	±0.17
50.00	96.3	±3.6	98.9	±0.0	96.5	±0.1	99.42	±0.04
25.00	98.7	±0.1	98.9	±0.1	97.3	±0.0	98.94	±0.22
12.50	73.4	±1.6	66.6	±2.6	96.9	±0.1	65.46	±2.66
6.25	42.6	±5.8	30.6	±2.2	43.9	±1.4	3.34	±2.58
3.13	11.6	±4.3	-1.1	±0.4	-2.1	±4.3	3.75	±4.47
1.56	0.4	±1.9	-2.0	±0.0	-8.8	±4.0	2.69	±6.68
0.78	-7.4	±8.1	-2.0	±0.1	-6.7	±2.9	1.05	±3.82
**IC_{50}**	**7.19±0.60**		**9.23±0.27**		**7.16±0.22**		**10.02±1.4**	

Table 2. Percentage of growth inhibition of Leishmania amazonensis with nucleotides (NT) and AHCC. No activity was shown, therefore IC_{50} could not be calculated.

Concentration (µg/mL)	AHCC		NT		1NT: 2AHCC		1NT:1AHCC		2NT:1AHCC		1NT:3AHCC		3NT:1AHCC	
**200**	13.09	±0.72	9.70	±1.09	14.80	±3.12	15.00	±2.34	17.13	±2.91	-1.78	±1.43	14.10	±3.1
**100**	13.56	±4.31	14.12	±0.73	15.63	±2.29	15.57	±6.53	8.78	±9.52	-0.51	±2.85	9.49	±2.4
**50**	10.42	±5.17	15.92	±2.01	6.05	±4.42	8.35	±2.72	-0.40	±5.03	-7.02	±6.95	-3.01	±2.1
**25**	-4.07	±1.05	-4.83	±2.45	-3.92	±6.99	-7.17	±5.78	-2.14	±3.24	-2.05	±2.35	-4.11	±6.4
**12.5**	-9.16	±7.27	5.30	±3.57	-10.54	±5.66	-8.44	±4.16	-9.87	±3.80	0.86	±2.65	1.06	±8.7
**6.25**	-8.59	±6.16	-16.43	±3.56	-17.63	±4.04	-18.17	±6.86	-11.40	±8.87	7.03	±2.00	7.13	±2.0
**3.12**	-27.10	±6.33	-26.52	±4.87	-24.56	±3.39	-17.28	±3.76	-8.46	±5.11	0.49	±2.66	6.38	±1.3
**1.56**	-20.51	±6.21	-23.33	±3.55	-11.54	±4.32	-6.66	±2.33	-8.72	5.18	-0.34	±2.22	5.75	±2.1

Table 3. Percentage of growth inhibition of Leishmania braziliensis with nucleotides (NT) and AHCC. No activity was shown, therefore IC_{50} could not be calculated.

Concentration (µg/mL)	AHCC		NT		1NT: 2AHCC		1NT:1AHCC		2NT:1AHCC		1NT:3AHCC		3NT:1AHCC	
**200**	3.16	±2.74	8.76	±1.20	-0.33	±1.25	-2.05	±0.61	1.56	±0.81	-12.24	±3.64	-15,.4	±6.78
**100**	2.90	±1.30	5.12	±0.93	-2.23	±0.88	-2.85	±0.98	-2.08	±3.87	-15.80	±15.20	-14.02	±5.32
**50**	1.73	±5.87	4.71	±3.69	-5.47	±1.27	-1.91	±1.23	-1.69	±3.11	-19.86	±11.96	-8.36	±11.78
**25**	-1.41	±1.10	-0.10	±1.95	-5.24	±0.90	-1.71	±0.70	1.70	±1.92	-7.34	±22.75	-3.44	±6.70
**12.5**	2.86	±1.74	-2.34	±3.21	-0.42	±3.83	4.92	±5.23	2.07	±1.95	-2.47	±16.80	2.77	±6.84
**6.25**	5.22	±0.88	-1.72	±3.23	3.15	±1.80	1.18	±4.09	0.73	±2.72	6.65	±27.62	8.92	±4.53
**3.12**	1.70	±4.71	3.86	±1.62	-0.40	±0.63	-0.27	±3.39	0.53	±1.90	-16.81	±6.58	7.36	±5.74
**1.56**	5.19	±0.32	6.46	±3.93	-1.43	±2.52	-1.27	±3.26	-5.86	±2.46	-5.62	±0.87	4.58	±1.46

Table 4. Percentage of growth inhibition of Leishmania donovani with nucleotides (NT) and AHCC. No activity was shown, therefore IC_{50} could not be calculated.

**Concentration (μg/mL)**	**AHCC**		**NT**		**1NT: 2AHCC**		**1NT:1AHCC**		**2NT:1AHCC**		**1NT:3AHCC**		**3NT:1AHCC**	
**200**	1.66	±0.36	4.76	±4.18	-1.72	±2.01	-0.54	±1.29	-4.04	±2.15	-4.75	±2.21	1.47	±2.78
**100**	1.01	±0.56	1.93	±6.44	-5.32	±1.59	-7.56	±1.73	-4.99	±2.60	-0.71	±1.30	-2.71	±1.88
**50**	-3.10	±3.16	1.00	±0.88	-5.46	±3.48	-4.88	±1.35	-6.61	±0.39	6.46	±2.91	2.50	±4.44
**25**	-1.43	±3.97	-0.89	±0.62	-5.27	±1.89	-6.82	±0.75	-3.95	±2.38	0.86	±3.21	0.10	±4.57
**12.5**	-0.51	±3.02	-1.19	±1.22	0.89	±5.85	-7.91	±1.85	-6.69	±3.45	4.65	±0.40	1.25	±1.84
**6.25**	-0.28	±1.52	-0.74	±1.33	-4.52	±2.81	-7.91	±1.43	-7.19	±3.99	1.57	±2.51	-0.25	±4.57
**3.12**	-1.34	±1.53	0.03	±1.45	-4.06	±4.86	-5.55	±3.37	-5.84	±2.28	0.96	±2.06	-1.09	±5.69
**1.56**	1.87	±2.30	0.49	±4.05	-3.48	±6.53	-2.91	±1.93	-8.73	±3.77	1.89	±2.58	-0.78	±1.06

Table 5. Percentage of growth inhibition of Leishmania infantum with nucleotides (NT) and AHCC. No activity was shown, therefore IC_{50} could not be calculated.

**Concentration (μg/mL)**	**AHCC**		**NT**		**1NT: 2AHCC**		**1NT:1AHCC**		**2NT:1AHCC**		**1NT:3AHCC**		**3NT:1AHCC**	
**200**	-2.88	±0.61	-0.71	±0.86	2.08	±5.41	-3.30	±4.03	0.64	±5.03	-1.36	±2.25	1.97	±2.48
**100**	-4.08	±0.86	-4.54	±1.19	-0.42	±1.44	-4.10	±1.35	-7.43	±1.72	-1.13	±1.17	1.42	±0.56
**50**	-7.47	±4.97	-0.62	±5.46	0.54	±2.20	-0.28	±3.73	-4.86	±1.92	0.25	±2.98	-0.09	±1.19
**25**	-1.97	±0.84	-3.64	±2.53	2.63	±2.78	-0.52	±1.82	-2.92	±0.78	1.03	±4.61	0.52	±0.87
**12.5**	-2.82	±0.95	-1.34	±2.54	-1.00	±2.05	3.88	±5.95	0.08	±2.08	-4.82	±0.99	-1.32	±0.82
**6.25**	-4.92	±1.61	-4.90	±0.08	-0.71	±1.75	-2.11	±3.33	-4.67	±1.64	-2.94	±0.57	-3.83	±2.11
**3.12**	-4.18	±1.03	-5.03	±0.67	-0.18	±2.36	-3.37	±1.20	-4.42	±0.40	-1.50	±2.80	-5.94	±0.94
**1.56**	-3.36	±1.00	3.01	±7.08	7.03	±5.81	2.57	±3.10	1.03	±1.90	-0.98	±1.44	-6.03	±0.97

In vitro amastigote susceptibility assays

Table 6. Percentage of growth inhibition of different Leishmania species with reference drug miltefosine.

Concentration (µg/mL)	*L. amazonensis*		*L. infantum*	
50.00	100.0	±0.0	99.24	±0.01
25.00	95.2	±4.2	99.23	±0.10
12.50	87.6	±6.3	99.83	±0.18
6.25	62.0	±12.4	97.28	±0.72
3.13	31.5	±9.2	77.95	±6.50
1.56	7.0	±12.2	52.64	±8.98
0.78	7.0	±12.2	47.88	±9.92
0.39	3.8	±6.5	0	±0.00
**IC_{50}**	4.68±1.1		2.65±0.40	

Table 7. Percentage of growth inhibition of Leishmania infantum with nucleotides (NT) and AHCC. No activity was shown, therefore IC_{50} could not be calculated.

Concentration (µg/mL)	AHCC		NT		1NT: 2AHCC		1NT:1AHCC		2NT:1AHCC		1NT:3AHCC		3NT:1AHCC	
**200**	0.84	±1.02	-0.14	±3.54	-7.18	±4.76	5.79	±7.85	1.74	±1.39	-6.19	±7.01	4.79	±6.60
**100**	2.71	±3.71	-0.47	±1.87	-6.32	±2.16	-5.09	±10.05	-6.53	±7.37	-6.01	±9.20	4.73	±11.00
**50**	1.75	±2.36	2.80	±7.27	-2.43	±1.81	7.94	±1.66	-1.94	±5.13	-5.81	±2.81	5.84	±4.19
**25**	0.77	±9.48	-6.68	±2.72	3.44	±4.81	7.93	±0.51	-5.86	±8.75	6.44	±8.18	8.50	±2.10
**12.5**	-3.92	±13.75	-2.00	±6.13	-9.18	±7.05	1.87	±6.48	0.77	±0.54	-6.51	±1.92	4.28	±1.76
**6.25**	1.02	±2.56	1.87	±4.36	-4.47	±4.25	6.11	±3.91	2.74	±4.67	-4.22	±6.65	6.33	±6.11
**3.12**	-1.85	±12.52	-5.30	±11.01	-12.61	±7.84	-1.20	±7.20	-5.48	±8.26	-7.47	±4.79	3.41	±4.40
**1.56**	-2.67	±7.92	-7.62	±5.96	-6.05	±1.22	4.69	±1.12	1.11	±2.61	-2.72	±5.08	7.68	±4.66

Table 8. Percentage of growth inhibition of Leishmania amazonensis with nucleotides (NT) and AHCC. No activity was shown, therefore IC_{50} could not be calculated.

Concentration (µg/mL)	AHCC		NT		1NT: 2AHCC		1NT:1AHCC		2NT:1AHCC		1NT:3AHCC		3NT:1AHCC	
**200**	-1.45	±1.04	-11.73	±7.73	-2.56	±8.54	-4.83	±8.73	-4.95	±1.42	-3.90	±7.79	-5.94	±7.34
**100**	0.47	±3.79	-6.26	±7.44	0.58	±2.28	-2.79	±2.96	-2.89	±1.89	-4.87	±4.18	5.77	±4.61
**50**	-2.83	±4.05	-5.24	±2.35	-0.22	±1.81	-2.43	±1.85	-6.46	±1.52	-3.53	±3.78	-4.76	±4.67
**25**	-3.84	±6.24	2.47	±3.41	0.92	±5.09	5.80	±6.79	-5.81	±3.68	0.40	±3.17	6.44	±1.08
**12.5**	-1.67	±6.42	-4.35	±6.27	-4.52	±5.37	-9.18	±7.21	-8.41	±1.46	-4.21	±1.63	-4.15	±2.77
**6.25**	-1.26	±2.61	-0.39	±4.46	-2.21	±4.25	-4.47	±4.35	-3.88	±4.77	-1.97	±8.81	0.49	±2.85
**3.12**	-7.68	±9.90	-10.04	±13.73	-2.10	±1.21	-3.18	±0.87	-12.67	±8.45	-5.14	±6.19	-0.40	±1.36
**1.56**	-2.71	±4.25	-8.94	±5.66	-3.76	±1.22	-6.05	±1.24	-5.63	±2.67	-0.50	±6.75	-0.37	±4.03
